# Public Health Adaptation to Climate Change in Canadian Jurisdictions

**DOI:** 10.3390/ijerph120100623

**Published:** 2015-01-12

**Authors:** Stephanie E. Austin, James D. Ford, Lea Berrang-Ford, Malcolm Araos, Stephen Parker, Manon D. Fleury

**Affiliations:** 1 Department of Geography, McGill University, Burnside Hall Building Room 705, 805 Sherbrooke Street West, Montreal, QC H3A 0B9, Canada; E-Mails: james.ford@mcgill.ca (J.D.F.); lea.berrangford@mcgill.ca (L.B.-F.); malcolm.araosegan@mail.mcgill.ca (M.A.); 2 Enteric Surveillance and Population Studies Division, Centre for Food-borne, Environmental and Zoonotic Infectious Diseases, Public Health Agency of Canada, 255 Woodlawn Road West, Unit 120, Guelph, ON N1H 8J1, Canada; E-Mail: stephen.parker@phac-aspc.gc.ca; 3 Environmental Issues Division, Public Health Agency of Canada, 255 Woodlawn Road West, Unit 120, Guelph, ON N1H 8J1, Canada; E-Mail: Manon.D.Fleury@phac-aspc.gc.ca

**Keywords:** climate change, adaptation, Canada, public health, adaptation tracking

## Abstract

Climate change poses numerous risks to the health of Canadians. Extreme weather events, poor air quality, and food insecurity in northern regions are likely to increase along with the increasing incidence and range of infectious diseases. In this study we identify and characterize Canadian federal, provincial, territorial and municipal adaptation to these health risks based on publically available information. Federal health adaptation initiatives emphasize capacity building and gathering information to address general health, infectious disease and heat-related risks. Provincial and territorial adaptation is varied. Quebec is a leader in climate change adaptation, having a notably higher number of adaptation initiatives reported, addressing almost all risks posed by climate change in the province, and having implemented various adaptation types. Meanwhile, all other Canadian provinces and territories are in the early stages of health adaptation. Based on publically available information, reported adaptation also varies greatly by municipality. The six sampled Canadian regional health authorities (or equivalent) are not reporting any adaptation initiatives. We also find little relationship between the number of initiatives reported in the six sampled municipalities and their provinces, suggesting that municipalities are adapting (or not adapting) autonomously.

## 1. Introduction

Climate change has been identified as a major risk to human health [1]. Climate-related health risks are already evident in events such as the 2003 European heat wave and flooding in Alberta, Canada in 2013, and demonstrate that high income countries such as Canada will be affected by the health effects of climate change [2,3]. In light of the risks posed by climate change, the Intergovernmental Panel on Climate Change (IPCC) calls for greater global action to reduce greenhouse gas emissions [4]. Yet, as the recent IPCC Fifth Assessment Report highlights, some degree of climate change and associated health impacts are inevitable, increasing the importance of adaptation [5]. Adaptation refers to policies, measures and strategies designed to reduce climate change impacts and support resilience [6], and in a health context is synonymous with prevention (*i.e.*, it seeks to prevent/minimize impacts), and may involve primary, secondary, and tertiary interventions [7,8,9,10]. While numerous opportunities for adaptation in a health context exist, managing the effects of climate change on human health has nevertheless been identified as a formidable challenge for both policy makers and health professionals [11].

Alongside changing precipitation patterns, the annual average surface temperature over Canada’s landmass has increased by 1.7 °C since 1948, a rate almost twice the global average, and by even more in Northern Canada, which is experiencing the most rapid climate change globally [12,13]. The health impacts of climate change affect Canadians through both direct exposures, such as injuries and death from extreme heat events, and through indirect exposures, for example by creating favourable conditions for outbreaks of infectious diseases or by negatively impacting socioeconomic conditions. Some health impacts will be felt throughout Canada, while others will be concentrated in certain regions [14]. Climate change will benefit Canadians’ health in some ways also, for instance by reducing cold-related mortality and providing economic benefits in some sectors, but overall the health burden is expected to increase [5,15].

The approach of various levels of government in Canada to adaptation has been described as a “multi-level mosaic”, loosely connected and emerging spontaneously [16]. Responsibility for adaptation remains unclear and is divided between the federal government, its 10 provinces and 3 territories [16]. The Government of Canada published the Federal Adaptation Policy Framework in 2011 which outlines the federal role in adaptation (generating and sharing information; building adaptive capacity; and mainstreaming adaptation policies) and provides criteria for federal departments and agencies to consider when identifying climate change adaptation priorities [17]. This framework is valuable and an important development, but does not identify how the federal government will work with other levels of government and does not suggest a direction for Canada as a whole to take in adapting to climate change, outside of the federal government; in the general scholarship, these have been identified as important components of government planning for adaptation [18,19,20]. Some climate change effects will be felt in localized areas of Canada, meaning much adaptation will also need to be implemented by municipal governments [21].

Despite the importance of adaptation in a public health context, to our knowledge there has not been a systematic examination of the current state of health adaptation across Canada. Health Canada and Natural Resources Canada, departments of the federal government, have conducted comprehensive vulnerability assessments which outline some examples of adaptation [14,22,23]. However, these assessments were not designed to characterize and compare adaptations at different jurisdictions, across regions, and over time. Reflecting this, in this study we systematically review Canadian health adaptation at the federal, provincial, territorial and local levels, examine their jurisdictional patterning, and outline gaps in health adaptation. Developing an understanding of how health adaptation is currently taking place in Canada, the work provides a baseline for future monitoring of adaptation progress. We begin the paper by outlining the risks posed by climate change on Canadians’ health, before presenting our methods and results.

## 2. Climate Change Risks for Canadian Public Health

**Figure 1:**
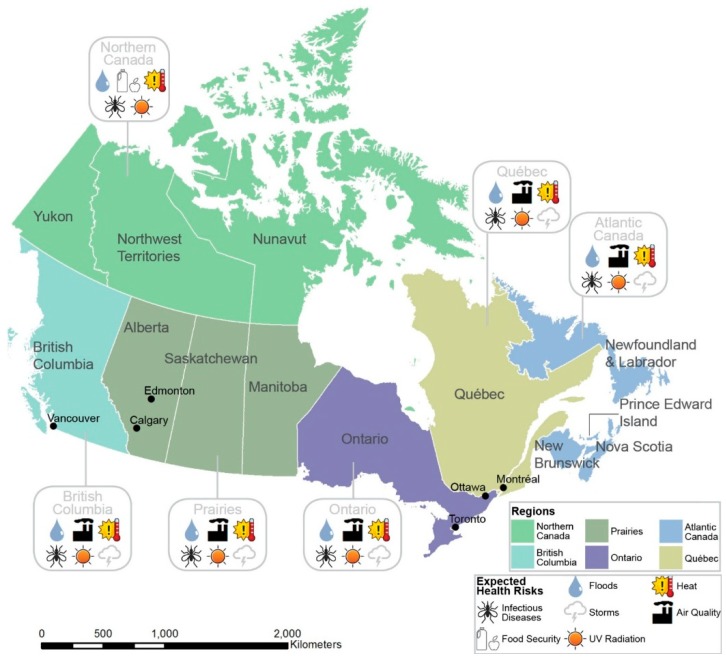
Expected health risks posed by climate change by region.

The projected impacts of climate change on Canadians’ health are diverse, complex, and incompletely understood for some health outcomes (see Figure 1). For instance, research has yet to reach consensus on the potential effect of climate change on hurricanes [24,25], severe thunderstorms, and the high winds and tornadoes associated with thunderstorms, all of which are climatic events pertinent to health in Canada [26,27]. Studies project freezing rain events are likely to increase in some Canadian regions, and decrease or remain the same in others [28,29]. The frequency and magnitude of floods have increased in the last century [30] and are projected to increase in the future in most—but not all—flood-risk areas [5,31,32,33].

During the 20th century the occurrence of cold nights, cold days and frost days decreased in Canada [34], along with the duration, frequency and intensity of cold spells [35,36]. Meanwhile, the occurrence of warm nights, warm days and summer days has increased and is expected to continue rising under future climate change scenarios [14,34]. Likewise, extreme heat events are projected to increase in intensity, frequency and duration [33,37]. The negative health effects of extreme heat events in Canada will be amplified by an aging population and the urban heat island effect, particularly in regions with high population density [37,38]. Typically there are higher cold-related mortality rates in Canada, but climate change may shift the balance by increasing summer mortality rates while cold-related mortality remains constant or decrease [15].

Climate change is expected to increase exposure to ultraviolet radiation (UVR) through UV dose, exposure duration and ozone depletion, particularly in northern Canada [39,40,41,42]. Each degree Celsius increase in temperature, for example, increases the effective UVR dose by about 2% [42]. While some exposure to UVR is beneficial to health, overexposure can pose serious risks [43,44,45]. Climate change is also expected to negatively affect air quality in urban areas by increasing production of summertime surface ozone and potentially also influencing particulate matter (PM) concentrations [46]. One study found the highest projected increases in ozone concentrations for Montreal, Toronto, Vancouver, Calgary, Edmonton and Winnipeg, as well as large increases in Alberta near the oil sands developments [14]. In Canada, PM impacts may increase or decrease with climate change, depending heavily on biogenic processes. Wildfires are currently responsible for one third of all PM emissions in Canada, and are increasing in frequency, duration and season length, contributing to heart and respiratory illnesses [33,47,48].

Food- and water-borne infections are likely to increase and may result in gastrointestinal illness and in some cases chronic health problems (*i.e.*, kidney failure) [14,48,49]. Some diseases such as acute gastrointestinal illness (AGI) have been associated with conditions that may be altered by climate change, such as higher temperatures and changing precipitation patterns, respectively [49]. Heavy precipitation events, projected to increase in many regions of North America, are strongly correlated to incidence of water-borne diseases [48].

Climate change is expanding the geographical range of tick populations (the vector for Lyme disease) northward in Canada, consequently also expanding the potential for Lyme disease [50,51]. Similarly, outbreaks of West Nile virus, transmitted by mosquito vectors, have been linked to conditions of mild winters, prolonged droughts and heat waves, conditions which are more likely to occur with climate change [52]. The prevalence and range of other viruses transmitted by mosquito vectors currently found in the United States, such as St. Louis encephalitis, Western equine encephalitis and Eastern equine encephalitis, could also be modified by climate change [14].

Communities in the Canadian Arctic face unique challenges and are particularly sensitive to the impacts of climate change [43]. This relates in part to the continued importance of subsistence harvesting to livelihoods, the prevalence of traditional food cultures, and dependence on the rapidly changing sea, lake, and river ice for transportation between communities and to hunting grounds. Socio-economic and health inequalities are also pronounced among northern Indigenous populations, and are expected to exacerbate the impacts of climate change [53]. Climate change has already been documented to be compromising food insecurity, enhancing danger of engaging in land-based activities, and compromising the ability to practice traditional cultural activities, with associated psychosocial consequences well documented [43,54,55].

Food systems in the Canadian Arctic are already being affected by the impacts of climate change on subsistence harvesting activities, and climatic changes are posing a threat to the food security of Northern populations by influencing animal availability, human ability to access wildlife, and the safety and quality of wildlife for consumption [23,56,57]. There have been few studies examining the effect of climate change on food security elsewhere in the country, however [58]. Canada is relatively food secure overall and is a food exporter, thus is expected to be less vulnerable to global yield fluctuations [33].

Climate change will have varied impacts on Canadians’ livelihoods, with implications for social gradients in health [33]. People with lower socioeconomic status are already exposed to multiple risk factors, including housing and neighbourhood quality, and adverse interpersonal relationships, converging in physical and psychosocial health consequences [59]. Groups that are already the most socially and economically disadvantaged are believed to be the most vulnerable to climate change, with vulnerability exacerbated and manifest through existing inequalities [11,60]. Definitions of key terms such as vulnerability and health risk are provided in Appendix A in the supplementary materials.

## 3. Methods

We employed systematic review methods to document and characterize adaptation initiatives being undertaken across Canada, with methods varying by, and adapted to, jurisdiction. The approach builds upon emerging research in the adaptation tracking field, which seeks to identify and characterize the current status of adaptation and measure progress over time [61,62]. To identify federal-level adaptation initiatives, we conducted a systematic web search, reviewed Canada’s sixth national communication (NC6) to the United Nations Framework Convention on Climate Change (UNFCCC) and conducted snowball searching. We developed the following search string tailored for Google web searches: *(“climate change” OR “global warming” OR “climatic change”) health (adaptation OR adaption OR ~adapting OR ~coping OR response OR ~responding) (Canada OR Canadian OR “gc.ca”).* We examined the first 30 search results, then examined every other result after the first 30 until we had reached 30 consecutive irrelevant results, adapted from Panic & Ford [63]. To track provincial and territorial health adaptation we used keyword searches on their respective Ministry of Health, Ministry of Environment, and Climate Change Department (where applicable) websites. At the local level we searched for evidence of health adaptation in Canada’s six largest municipalities (population >1 million inhabitants) and in each municipality’s encompassing regional health authority (or equivalent). Under Canada’s health care system regional health authorities are responsible for health care delivery at the sub-provincial or sub-territorial level. To track municipal and regional health authority adaptation initiatives we used keyword searches on their respective websites. We conducted searches in French in jurisdictions where relevant. We developed inclusion and exclusion criteria for health adaptation documents (see Appendix B in the supplementary materials). All documents included in our study are listed in Appendix C in the supplementary materials.

We documented details of discrete adaptation initiatives found in included documents, including year, implementing body, adaptation type, adaptation stage, health vulnerability addressed, completion status, and consideration of vulnerable groups. Adaptation type refers to the following six adaptation categories adapted from Biagini *et al.* [64] (Table 1): capacity building; management, planning and policy; practice and behavior; information; warning and observing systems; and vulnerability assessments. As per Lesnikowski *et al.* [65], adaptation *stage* refers to either: groundwork actions which build adaptive capacity, prepare the conditions for adaptation, or enable adaptation actions; or adaptation actions which indicate action has actually been taken to reduce a population’s health vulnerability or increase resilience. Groundwork actions can be further classified as information and vulnerability assessment adaptation types, while adaptation actions are warning and observing systems, and practice and behaviour initiatives. Capacity building and management, planning and policy adaptation types could be classified as either groundwork or adaptation actions, depending on the characteristics of the discrete initiative.

**Table 1:** Health adaptation initiatives included, by adaptation type.

Adaptation Category	Description	Examples of Actions in Category
*Capacity Building*	Developing human resources, institutions, and communities, equipping them with the capability to adapt to climate change.	Training, best practices guidebooks, frameworks, public outreach and education, and dissemination of information to decision makers/stakeholders
*Management, Planning and Policy*	Incorporating understanding of climate science, impacts, and vulnerability and risk into government and institutional planning, management, policies and regulations.	Developing an adaptation plan, creating new bureaus or departments, bolstering emergency management plans for anticipated climate change impacts, and implementing legislation.
*Practice and Behaviour*	Revisions or expansion of practices and on the ground behaviour that are directly related to building resilience.	Eradication of mosquitoes, cool refuges, public drinking water developments, and increasing tree canopy.
*Information*	Systems for communicating climate information to help build resilience towards climate impacts (other than communication for early warning systems).	Decision support tools, communication tools, data acquisition efforts, and digital databases.
*Warning or Observing Systems*	Implementation of new or enhanced tools and technologies for communicating weather and climate risks, and for monitoring changes in the climate system.	Air quality indexes, infectious disease surveillance, and UV indexes.
*Vulnerability Assessment*	Assessment of projected health impacts and risks associated with future climate change.	Health impacts of climate change assessment, impacts of climate change on cities and rural communities, and health impacts of climate change on indigenous populations.

Note: Adapted from Biagini *et al.* [64].

Finally, we classified initiatives as addressing health risks identified by the IPCC (see Appendix A in Supplementary Material) [5]. For documents to be included in our analysis, they had to be a governmental document or webpage and report planned, in progress or completed adaptation initiatives addressing the health impact of climate change on humans. We collected data in a Microsoft Excel spreadsheet and performed descriptive statistics. To examine whether health adaptation is jurisdictionally patterned, we compared adaptation between jurisdictions. We also compared adaptation of municipalities and regional health authorities (or equivalent) in encompassing provinces to explore the effect of provincial and territorial adaptation on municipal adaptation. Based on the information available in academic literature and in the vulnerability assessments conducted by Health Canada and Natural Resources Canada [14,22,23], we identified the projected health impacts of climate change in Canadian provinces and territories, outlined in Section 2. As part of our analysis we then examined what percentage of the identified projected health risks are addressed by provinces and territoriesin their adaptation initiatives available online.

Our ability to identify adaptation initiatives was limited by how and if they were reported, noting we only capture explicitly and publicly reported adaptations. This represents a proxy sample rather than an exhaustive list of all adaptation actions. Whether jurisdictions choose to recognize and report activities related to adaptation is significant in itself as a potential reflection of the extent to which adaptation action and recognition is prioritized.

## 4. Results

### 4.1. Federal Health Adaptation Initiatives

**Figure 2:**
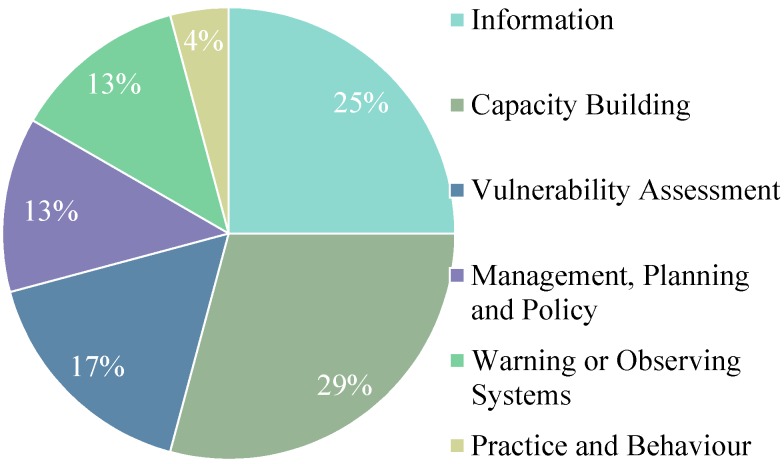
Percentage of federal health adaptation initiatives by adaptation type.

**Figure 3:**
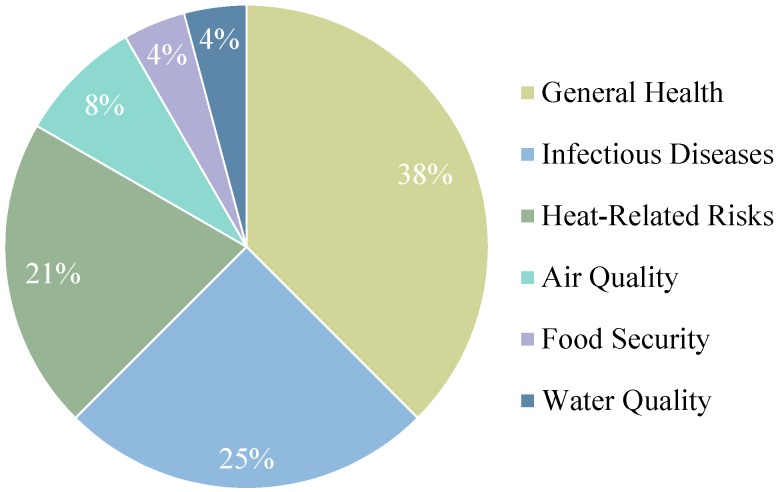
Percentage of federal health adaptation initiatives by health risk.

Canadian federal health adaptation initiatives emphasize capacity building and information type initiatives, addressing infectious diseases, heat-related risks or general health risks (Figure 2 and Figure 3). Contributing to the large number of capacity building initiatives is a series of heat risk guidelines and guidebooks prepared by Health Canada for public health officials, emergency management officials, health care workers and health administrators [66,67,68]. Meanwhile, most information initiatives reflect research conducted by the Public Health Agency of Canada that focuses on food and water safety and vector-borne diseases [69]. Both Health Canada and Natural Resources Canada have completed national vulnerability assessments (see Appendix D in Supplementary Material for descriptions of federal departments) [14,22,23]. Some of these health adaptation initiatives were implemented as part of the multi-departmental Adaptation Theme of the federal government’s Clean Air Agenda. Canada’s Federal Adaptation Policy Framework does not discuss any specific adaptation initiatives. The continuation and further expansion of health adaptation programs and funding availability depend on the priorities of individual departments and upper-level government. A list of all federal, provincial, territorial and municipal adaptation initiatives by type is shown in Table 2, and an Excel spreadsheet detailing all discrete adaptation initiatives is available in in the Supplementary Material (Appendix E).

The majority of federal health adaptation initiatives reflect groundwork initiatives, preparing the conditions for adaptive action. These groundwork initiatives include guidebooks, decision-making toolkits, vulnerability assessments and research. Federal adaptation *actions* (as opposed to groundwork initiatives) are primarily alert and response systems to air quality, infectious diseases and heat event risks. Half of the federal adaptation initiatives reviewed explicitly state consideration of vulnerable groups, particularly those addressing heat-related health risks.

### 4.2. Provincial and Territorial Health Adaptation Initiatives

All Canadian provinces and territories have publically accessible climate change adaptation plans except Alberta and Saskatchewan (Table 3). The median number of health adaptation initiatives implemented in Canadian provinces or territories is three initiatives, with Quebec reporting the highest number (31 initiatives). Newfoundland & Labrador report six initiatives, the next highest number of initiatives found online.

**Table 2:** Health adaptation initiatives included, by adaptation type.

Adaptation Category	Description	Federal Initiatives	Regional, Territorial and Provincial Initiatives	Municipal Initiatives
*Capacity Building*	Developing human resources, institutions, and communities, equipping them with the capability to adapt to climate change.	Climate Change Adaptation Program (AANDC) Climate change infections disease toolkit (PHAC) Heat alert and response systems best practices guidebook (HC) Heat risk adaptation guideline for public health and emergency management officials (HC) Heat risk technical guide for health care workers (HC)	Increase awareness about the health risk factors of climate change through legislation and awareness activities (ON) Raise public awareness of Lyme disease (ON) Training sessions for public health, clinic service and civil protection personnel concerning infectious diseases and emerging health problems (QC) “Changements climatiques: vulnérabilité et adaptation des immeubles” guide for health network administrators (QC) Support the training of specialized teams and implementing financial incentive measures to help eliminate urban heat islands in municipalities (QC) Enhancement of information and training tools and methods intended for the general public, organizations, medical staff and elected officials (QC) Launch of “Mon climat, ma santé” website to disseminate accessible information (QC) Raise the profile of climate change and health among public health professionals (NL) Hold conference on climate change and health to raise the profile of the impacts of climate change on public health (NL) Assist communities in developing emergency response plans (YT)	“Be Cool” communication campaign (Vancouver) Toronto food strategy (Toronto) Climate change and health equity workshop (Toronto)
*Management, Planning and Policy*	Incorporating understanding of climate science, impacts, vulnerability and risk into government and institutional planning, management, policies and regulations.	Pilot alert and response system to heat events (HC) Pilot alert and response systems to infectious diseases (PHAC) Creation of Water, Air and Climate Change Bureau (HC)	Increase awareness about the health risk factors of climate change through legislation and awareness activities (ON) Climate Change Adaptation Plans (NL, NS, ON, PE, QC, NU, YT) Catastrophe planning (PE) Coastal communities adaptation to coastal erosion (QC) Establishment of expert panels to assess measures needed for climate change adaptation (QC) Intervention plan to protect populations from West Nile virus (QC) Use flooding research results to map restricted development zones, prepare regulations and civil security plans (QC) Revise emergency plans (QC) Voluntary standard to plan public parking lots to reduce urban heat islands (QC) Requiring Regional Emergency Heat Wave Strategies (QC) Air Quality Policy (promoting local and regional air quality management) (QC) Elaborate legislation governing land-use planning and urban planning to prevent the appearance of new urban heat islands (QC) Working with municipalities to establish local emergency management to prepare for extreme weather events (MB) Increasing the speed and effectiveness of local and regional emergency response measures (MB) Work towards fully integrated emergency management system that takes climate change impacts into account (NB) Incorporate climate change considerations into emergency planning, working with all stakeholders (NU)	Climate change adaptation plan (or related document) (Toronto, Vancouver, Ottawa, Edmonton) Emergency preparedness (Ottawa, Edmonton, Montreal) Heat alert system and hot weather response plan (Toronto) Expanding extreme hot weather preparedness work program (Vancouver) Contingency plans for the emergence of West Nile virus (Montreal) Contingency plans for the increase in heat waves (Montreal) Shade policy and guidelines (Toronto)
*Practice and Behaviour*	Revisions or expansion of practices and on the ground behaviour that are directly related to building resilience.		Air Action Plan (BC) Increase awareness about the health risk factors of climate change through legislation and awareness activities (ON) Requiring regional emergency heat wave strategies (QC) Upgrade the physical buildings of health establishments to allow them to function independently over extended periods of time (QC) Personalized, automated warning systems (by telephone and internet) for vulnerable individuals (QC) Enhance assistance and psychosocial support measures following disasters (freezing rain, high tides, flooding) (QC)	Heat island controlling methods (Ottawa, Toronto) West Nile virus control measures (e.g., spraying at larval state) (Ottawa) Develop cool refuges (Vancouver, Toronto) Expand public access to drinking water (Vancouver) Include instances for mould in new online rental databases (Vancouver) Target green space and trees in hot areas (urban heat islands) (Vancouver)
*Information*	Systems for communicating climate information to help build resilience towards climate impacts (other than communication for early warning systems).	Assessment of the burden of AGI and adaptation to climate change in the Canadian North (PHAC) Climate change scenarios program (Part of CAA) (EC) Evaluation of the impacts of climate change on food and water safety and public health outcomes (PHAC) Projected burden of Lyme disease in Ontario (PHAC) Public health and water-borne illness research tool (PHAC) Creation of Water, Air and Climate Change Bureau, responsible for building knowledge (HC)	Flood risk mapping (BC, NB, NL) Analyze incidence and distribution of GI disease in at-risk populations and risk factors associated with climate change (QC) Development of an interactive atlas of health vulnerabilities to climate change (QC) Estimate and model future levels of smog (QC) Identification of urban areas vulnerable to intense heat (QC) Quebec flooding research (QC) Research on historical morbidity and hospital visits according to historical temperatures and simulated for estimated future temperatures (QC) Water management feasibility study (QC) Evaluate relevance and feasibility of establishing a monitoring system focusing on the psychosocial impact of extreme weather events (QC) Drinking water supply vulnerability analyses (NU) Monitoring health trends to identify and develop responses (YT) Short and long term epidemiological monitoring system for physical and psychosocial health problem related to extreme climate events (QC)	Spatial heat vulnerability assessment (Toronto) Urban heat island research (Toronto) Assess cooling capacity of cool refuges (Vancouver) Explore the potential for cooling rooms in non-market housing (Vancouver) Research transportation to cool facilities for those in need (Vancouver) Urban heat island mapping (Vancouver) Vulnerable coastal populations mapping (Vancouver)
*Warning or Observing Systems*	Implementation of new or enhanced tools and technologies for communicating weather and climate risks, and for monitoring changes in the climate system.	Air Quality and Health Index (HC, EC) Pilot alert and response system to heat events (HC) Pilot alert and response system to infectious diseases (PHAC)	Improved infectious disease surveillance (NL, NS, QC) Air quality index (NS, ON) Monitoring, surveillance and warning system for intense heat (QC) SUPREME System—Monitoring and surveillance system for public health (QC) Flood alert systems (NL)	Heat alert (Ottawa, Toronto, Vancouver) Set up/enhance ambient air quality monitoring program (Ottawa) Air quality health index (Ottawa, Toronto) Flood warning forecasting (Toronto)
*Vulnerability Assessment*	Assessment of projected health impacts and risks associated with future climate change.	Assess vulnerabilities and health impacts of climate change among Northern/Inuit populations (HC) Assist Northerners in assessing key vulnerabilities and opportunities (Part of CAA) (AANDC) Canadian assessment of vulnerabilities and adaptive capacity (HC) From Impacts to Adaptation: Canada in a Changing Climate 2007 (NRC)	Adaptability of Prairie Cities: The Role of Climate Current and Future Impacts and Adaptation Strategies (PARC)	

AANDC: Aboriginal Affairs and Northern Development Canada; PHAC: Public Health Agency of Canada; HC: Health Canada; EC: Environment Canada; NRC: Natural Resources Canada; BC: British Columbia; ON: Ontario; QC: Quebec; NS: Nova Scotia; NL: Newfoundland & Labrador; PE: Prince Edward Island; YT: Yukon; NU: Nunavut; NT: Northwest Territories; PARC: Prairies Adaptation Research Collaborative (Alberta, Saskatchewan and Manitoba Provinces).

Alberta and Saskatchewan do not report any health adaptation initiatives individually, but combined with Manitoba, their governments are part of the Prairies Adaptation Research Collaborative (PARC) which has implemented three general health initiatives: a vulnerability assessment feasibility report, a study of adaptability of Prairie cities, and an elders’ forum on climate change [70,71,72].Since these three regional initiatives were implemented jointly by all three provinces, for analysis our results show Alberta and Saskatchewan province as having implemented one initiative each. PARC is a good example of how provinces or territories can cooperate to adapt to regional climate change impacts.

**Table 3:** Population size, adaptation plans and health initiatives in Canadian provinces and territories.

Province/Territory	Population [73]	Adaptation Plan Available	Health Adaptation Initiative(S) Reported
Alberta	4,025,100	-	√ ^a^
British Columbia	4,582,000	√	√ ^b^
Manitoba	1,265,000	√	√
New Brunswick	756,100	√	√
Newfoundland &Labrador	526,700	√	√
Northwest Territories	43,500	√	√ ^b^
Nova Scotia	940,800	√	√
Nunavut	35,600	√	√
Ontario	13,538,000	√	√
Prince Edward Island	145,200	√	√
Quebec	8,155,300	√	√
Saskatchewan	1,108,300	-	√ ^a^
Yukon	36,700	√	√

^a^ Alberta, Manitoba and Saskatchewan have jointly implemented three regional initiatives, but Alberta and Saskatchewan have not implemented any individually; ^b^ British Columbia’s adaptation plan does not include health initiatives, but health adaptation initiatives were identified in other documents or webpages.

**Figure 4:**
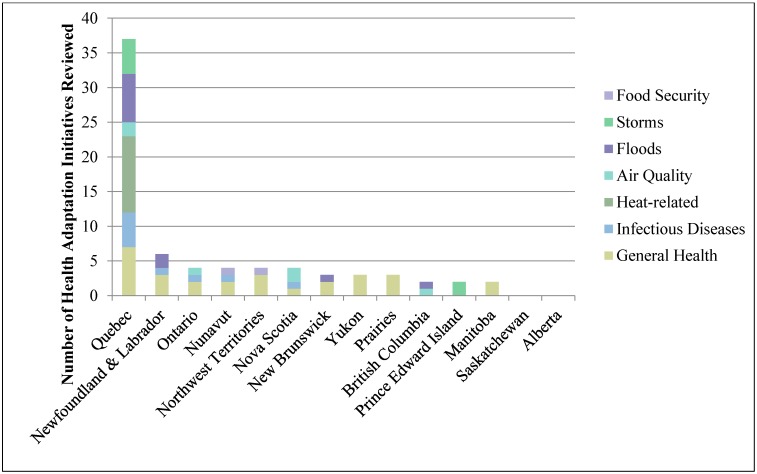
Types of climate change risks addressed by provincial and territorial health adaptation initiatives.

Based on the information available online, Quebec addresses almost all of the identified climate change health risks relevant to the province. Most other provinces and territories report addressing fewer than half of the identified health risks. The health risks addressed by each province or territory are shown in Figure 4. Aggregated to the regional level (region classification is shown in Figure 1), Quebec reports addressing the highest percentage of regional risks, followed by Atlantic Canada. Meanwhile, Northern Canada, Ontario, British Columbia and the Prairies region each address fewer than half of the identified regional health risks in the health adaptation initiatives available online.

**Figure 5:**
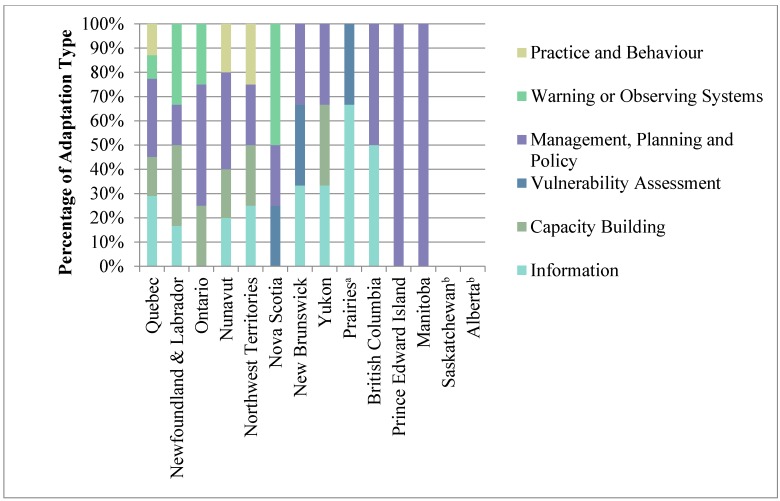
Percentage of health adaptation types by province or territory. Adapted from Biagini *et al.* [64]. ^a^ The three Prairie provinces (Alberta, Saskatchewan and Manitoba) have implemented three joint regional initiatives.; ^b^ Saskatchewan and Alberta do not report implementing any health adaptation initiatives other than the three joint Prairie provinces initiatives.

Of the provinces and territories reporting health adaptation initiatives, all include initiatives with elements of *management, planning and policy*, and most include initiatives with some *information* or *capacity building* aspects (Figure 5). Only the Northwest Territories and Nova Scotia have specifically targeted vulnerability assessments available online for their jurisdiction. All provinces and territories reporting initiatives specify considering vulnerable populations, including the elderly, the very young and Indigenous populations in their initiatives, except British Columbia, Prince Edward Island, and New Brunswick. Most provinces and territories reporting adaptation initiatives have a combination of both groundwork actions and adaptation actions. Most of the health adaptation initiatives reviewed have been implemented or are ongoing.

### 4.3. Adaptation Initiatives in Municipalities or Regional Health Authorities

Among Canada’s six largest municipalities, only Toronto and Vancouver have adaptation plans available. Montreal, Ottawa and Edmonton report health adaptation initiatives in other documents or on websites. Calgary is the only large municipality that does not report any health adaptation initiatives online (Table 4). Though regional health authorities are responsible for sub-provincial or sub-territorial health care delivery, none of the six sampled regional health authorities report any health adaptation initiatives or discuss climate change on their websites. Nonetheless, Vancouver reports a partnership on one initiative with its regional health authority, Vancouver Coastal Health.

**Table 4:** Adaptation plans and initiatives in Canada’s largest municipalities.

Municipality	Adaptation Plan Available	Health Adaptation Initiative(s) Reported
Calgary, AB	-	-
Edmonton, AB	-	√
Montreal, QC	-	√
Ottawa, ON	-	√
Toronto, ON	√	√
Vancouver, BC	√	√

Toronto and Vancouver emerged as leading municipalities in public health adaptation to climate change based on our analysis of publically available information. Both are the only municipalities reviewed to include plans for monitoring and evaluation of adaptations implemented. The majority of Toronto’s health adaptation initiatives were adaptation actions that addressed extreme heat, but others addressed air quality, food safety and security, UV radiation, floods and general health vulnerabilities. Toronto’s adaptation initiatives are highly varied, including actions such as providing cooling centres during extreme heat events, and implementing a heat alert system and hot weather response plan.

Similarly, the majority of health adaptation initiatives implemented by the City of Vancouver address heat-related climate change health risks. Almost half of the initiatives consider vulnerable groups, primarily homeless and low-income populations.

Edmonton, Montreal and Ottawa do not have publically available or reported adaptation plans, but they describe a small number of health adaptation initiatives in other documents or on their websites. These initiatives are predominantly management and planning adaptation actions (e.g., contingency plans for the increase in heat waves, emergency response plans for severe weather events), while Ottawa also reports some initiatives aimed at altering practice and behavior (e.g., urban heat island controlling methods, West Nile disease control measures).

### 4.4. Emerging Leaders and Progress among Canadian Provinces and Territories

**Figure 6:**
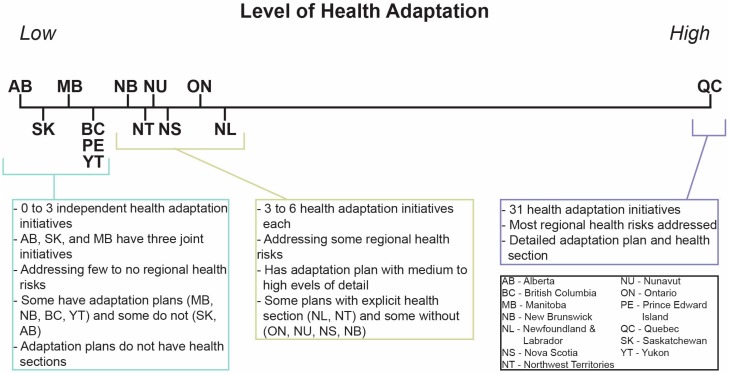
Level of health adaptation by province or territory ^a^. ^a^ The provinces and territories have been ranked qualitatively by number of health adaptation initiatives, percentage of regional health risks addressed, having a climate change adaptation plan, and the quality of the adaptation plan. See Appendix F in supplementary material for further details on the development of this figure.

Based on our analysis of publically available information, Quebec emerged as the leader in provincial and territorial health adaptation (Figure 6). The province has a comprehensive climate change adaptation plan with a detailed health section, addresses most regional health risks and has a diverse assortment of adaptation types. Quebec is bolstered by being home and partner to Ouranos, a consortium on regional climatology and adaptation practitioners, which contributes to the knowledge base on impacts, vulnerabilities and adaptation in the province. Similar to the United Kingdom Climate Impacts Program (UKCIP), Ouranos functions as a boundary organization that seeks to bridge science and policy to increase engagement of decision-makers, build adaptive capacity and create a network of knowledge [74]; such boundary organizations have been identified as an important component of institutional development for promoting readiness for adaptation [18,75]. Ouranos emerged in response to several extreme weather events (1998 ice storm and 2000 flooding in Quebec, and the European heat wave in 2003) which incited support for climate change research [76]. Gosselin *et al.* suggest adaptation to the health impacts of climate change in Quebec is taking place because of stable financing (money from the province’s carbon tax funds its climate change action plan), willingness for transdisciplinary collaboration, openness to public health innovation, and the knowledge provided by Ouranos on the current and projected impact of climate change in the province [76].

The remaining provinces and territories, based on publically available documents, are in the early stages of adaptation. Figure 6 shows the level of health adaptation in Canadian provinces and territories ranked qualitatively by number of initiatives found online, addressing identified regional health risks, having an adaptation plan with a health section, level of detail, and availability of climate change information on health department websites. A middle-pack of provinces and territories emerges, consisting of New Brunswick, Newfoundland & Labrador, the Northwest Territories, Nova Scotia, Nunavut, and Ontario. These provinces and territories each report implementing three to six health adaptation initiatives, but vary in levels of detail, comprehensiveness and risks addressed. Lastly, Alberta, British Columbia, Manitoba, Prince Edward Island, Saskatchewan, and Yukon have each reported three or fewer health adaptation initiatives independently (Alberta, Saskatchewan and Manitoba have jointly implemented three initiatives), address few or none of the identified regional health risks, and provide limited detail.

### 4.5. Jurisdictional Patterning of Adaptation

**Figure 7:**
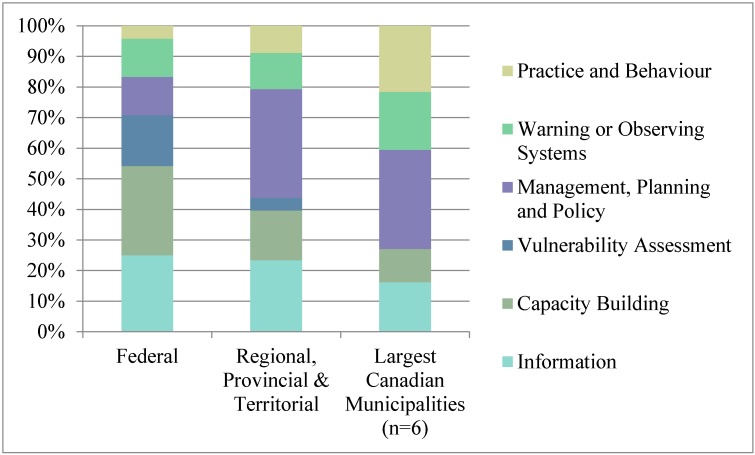
Average percentage of adaptation type by jurisdiction.

**Figure 8:**
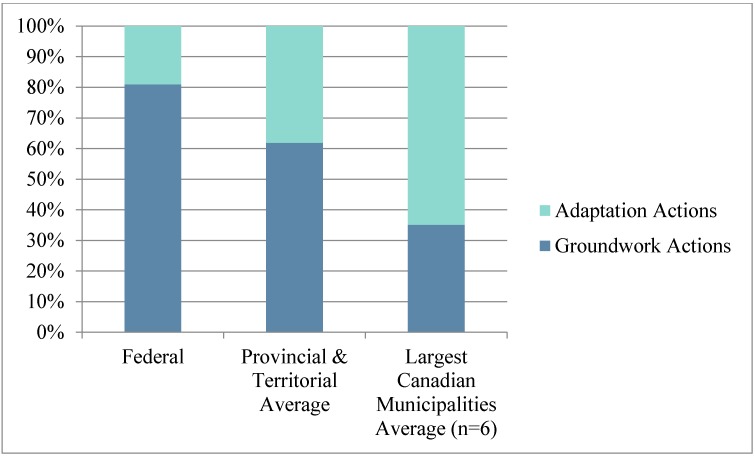
Average percentage of groundwork initiatives and adaptation actions by jurisdiction.

Canadian federal action on health adaptation largely consists of setting the stage for sub-national adaptation through *groundwork* capacity building and knowledge development. Meanwhile, local adaptation is operational, focusing on planning and changing practice and behavior “on the ground” (Figure 7 and Figure 8).

Divergent jurisdictional strategies are important here, since an adaptation initiative successful at one scale may not be considered successful at another [77]. Federal governments are best suited to intervene with public policy on issues affecting whole populations [77]. Some Canadian federal initiatives address specifically Northern populations which fall under the jurisdiction of the federal government, as dictated by Canada’s Constitution [78]. Sub-national governments meanwhile are often more flexible and can make and implement decisions more quickly [79]. Municipal governments have impetus for adaptation, since the benefits of these adaptive actions benefit them directly, and as key actors in adaptation need climate resources and decision support [80]. Current health adaptation to climate change reflects the jurisdictional responsibilities for health mandated by Canada’s Constitution, and the federal government’s role in adaptation outlined in Canada’s Federal Adaptation Policy Framework [17,78].

Despite these trends and the need for support, the influence of higher-level governments on municipal adaptation planning and implementation is not clear based on the publically available information reviewed here. Local governments are not entirely autonomous actors in adaptation and can be assisted or constrained by higher-level governments [77,80]. For example, changes in Chinese municipal government adaptation agendas are heavily influenced by national government incentives [81]; in contrast, where federal climate change action is limited such as in the United States, local governments may have the opportunity to be more entrepreneurial, innovative and autonomous in their adaptation strategies [80,82]. Based on our review, large Canadian municipalities seem to follow the latter pattern, where some cities such as Toronto and Vancouver have committed to adaptation, while others including Ottawa, Montreal, Edmonton and Calgary report doing little to nothing. Provincial adaptation also does not appear to exert a strong influence on municipal adaptation: Quebec is a leader in provincial adaptation, while Montreal, Quebec’s largest city, reported few health focused adaptations; British Columbia is a laggard among Canadian provinces, but its largest city Vancouver is progressing autonomously.

Assigning responsibility to jurisdictions for managing the risks of climate change impacts and financing adaptation is a major challenge in such cross-jurisdictional issues [83]. In contrast to the Canadian context where the responsibility for health and environment are primarily devolved to the provinces, the adaptation responsibilities of central government are clearer in other countries with different jurisdictional structures, such as Belgium and the United Kingdom [84,85]. These issues of scale and allocation of resources are difficult to resolve in unchartered cross-jurisdictional waters, and have been identified as barriers to adaptation in the broader scholarship [75,86,87,88,89].

## 5. Discussion

### 5.1. Gaps in Adaptation

The National Adaptation Policy Framework serves as Canada’s national adaptation strategy, and while an important development it does not have action points, lacks visibility and is not intended as an implementation framework, with implications for its potential role as a coherent adaptation strategy [90,91]. The Framework is very similar to Australia’s document “Adapting to Climate Change in Australia: An Australian Government Position Paper” which outlines the Australian’s government vision for, and role in, adaptation, including sub-national governments [92]. National adaptation plans of other OECD countries such as Belgium, Germany, Ireland, Switzerland and the United Kingdom however, go further, detailing the government’s general adaptation approach, specific adaptation options, and which departments or levels of government are involved (often divided sectorally). It is noteworthy here that Canada’s jurisdictional structure is markedly different from that of many Western European countries, inhibiting federal jurisdiction to engage in top-down guidance. Nonetheless, commentary on Canada’s federal role in adaptation has emphasized the need for a cross-cutting adaptation plan or strategy that will coordinate and clarify jurisdictional and sectoral roles and responsibilities in climate adaptation [83,93,94], with such frameworks also serving to institutionalize adaptation at sub-national scales [93].

Federal departments such as Health Canada and the Public Health Agency of Canada (PHAC) have examined the risks posed by climate change to Canadians and have begun to examine opportunities for adaptation; these represent important developments, and Canada has been regarded as a leader in examining vulnerabilities in a health context [63]. Climate change has also received increased emphasis within Canada’s federal agencies responsible for health: since 2007, for example, both Health Canada’s Climate Change and Health Office and climate change and health activities within the Public Health Agency of Canada expanded their full-time staff. However, further coordination and prioritization of climate change at the national level will be beneficial to develop and promote comprehensive climate and health programming. To this end, the Canadian government received significant criticism for a lack of leadership on adaptation issues by the Canadian Commissioner of the Environment and Sustainable Development [94], and similarly for poor performance in climate change policy and mitigation more generally [95].

Provinces and territories need vulnerability assessments to plan appropriate adaptation strategies [96]. Currently only the governments of Northwest Territories and Nova Scotia have independently conducted vulnerability assessments available online targeted to their specific jurisdiction and health. Natural Resources Canada conducted a regional vulnerability assessment, but the scale, level of detail, links between health and climate change, and overall relevance for adaptation planning varies by chapter [23].

While there is an increasing amount of scholarship examining the effect of climate change on some sub-populations believed to be highly sensitive to the effects of climate change (e.g., northern populations such as the Inuit) [6,43,56,97], other vulnerable populations are not consistently being targeted, such as First Nations, the elderly, children, socially disadvantaged, mentally ill, or chronically ill [5,97]. Vancouver succeeds in both identifying vulnerable groups within its jurisdiction and implementing tailored adaptation initiatives, targeting low-income populations [98]. Health Canada also identifies the risks and challenges for low-income populations in its extreme heat events toolkit for public health and emergency management officials, but does not offer any further adaptive responses tailored to this population [66]. No other jurisdictions consider low-income populations in adaptation initiatives reviewed here.

### 5.2. Future Health Adaptation

In light of the gaps identified in Canadian health adaptation, we suggest several steps to advance on adaptation in a health context:

- Development of a national adaptation research consortium, or further regional consortiums. A national or regional research consortium would serve to coordinate research activities and act as a boundary organization to link research and policy [18], as Ouranos does for Quebec and the National Climate Change Adaptation Research Facility (NCCARF) does for Australia. Development of Ouranos in the province of Quebec has helped to characterize and quantify the impacts of climate change on human health in Quebec and provide a solid knowledge base for subsequent Action Plans [76]. Similarly, the Australian government established the NCCARF to coordinate and manage climate change adaptation research. Evaluations of NCCARF have found that the Facility’s activities have improved Australia’s capacity to develop the knowledge necessary for effective adaptation decision-making [99].
- Development of provincial and territorial vulnerability assessments to guide sub-national health adaptation. Before making an adaptation plan and implementing adaptation actions, the first steps in adapting to climate change are understanding current and future climate vulnerability [96]. Sub-national governments will be able to make informed adaptation decisions that are context-appropriate and an efficient use of resources with access to detailed vulnerability assessments.
- Development of adaptation guides for regional health authorities, similar to the series of extreme heat events guidebooks developed by Health Canada [66,67,68]. Developing an adaptation guide could serve as a tool for regional health authorities to identify the necessary steps and considerations for adaptation. Some of the health effects of climate change will be felt locally, thus regional health authorities (or equivalent) must make adaptation to climate change a priority [21].
- Regular meetings of representatives from all sectors involved in federal, provincial and territorial health adaptation in Canada. Such meetings are important for coordinating activities, and working towards a more comprehensive national health adaptation strategy [18,20].
- Regular meetings between provinces and territories, and between municipalities and regional health authorities, to exchange knowledge and experience, such as in Switzerland where cantons are encouraged to exchange procedures and experiences during heat waves [100].
- Ensuring adaptation best practices such as consideration of vulnerable groups and monitoring and evaluation are being followed in future adaptation programming, guided by existing adaptation best-practice guides such as UKCIP’s Adaptation Wizard [101].

## 6. Conclusions

Health adaptation to climate change in Canada varies significantly by jurisdiction. Federal adaptation initiatives are primarily groundwork initiatives emphasizing capacity building and generating and sharing knowledge to support sub-national adaptation. This approach is congruent with the strategy outlined in Canada’s Federal Adaptation Policy Framework [17] and with Canada’s constitution which devolves responsibility for health and environment to the provinces and territories [78]. Sub-national adaptation focuses progressively less (between provincial/territorial and local adaptation) on information and capacity building initiatives, and progressively more on warning or observing systems and initiatives changing practice and behavior. Management, planning and policy is most heavily emphasized at the provincial and territorial jurisdiction.

The level of health adaptation also varies strongly within levels of jurisdiction. The province of Quebec reports online more than five times as many health adaptation initiatives as the next province or territory, addresses to some degree almost all identified health risks in the province and has a comprehensive adaptation plan. Meanwhile, the rest of Canada’s provinces and territories are still in the early stages of adaptation, based on publically available information. Similarly, the cities of Toronto and Vancouver both show commitment to adaptation and a long-term vision, while Canada’s next four largest municipalities report little to no evidence of health adaptation. Though not explored in this study, rural Canadian communities will face challenges distinct from those of their urban counterparts, including adaptive capacity, risk perception, and dependence on natural resources [102].

Variation in health adaptation between provinces and territories and between municipalities is inconsistent with risks posed to varying jurisdictions. Recent extreme events such as the Alberta floods in 2013, for instance, which caused the evacuation of over 100,000 people, demonstrate the magnitude of risks facing provinces and municipalities. The Alberta provincial government, Calgary and other municipal governments have been criticized for a lack of preparation and planning for climatic extremes [103,104]. Unaddressed, these jurisdictional differences may contribute to regional differences in the health impacts of climate change on populations. Federal guidance to assist such provinces and territories is important in this context.

Canada has the capacity to adapt, with the necessary wealth, technology, institutions, social organizations and skills. However, as Burton [105] notes in his concluding chapter to the Canadian National Assessment on climate change impacts and adaptation a spontaneous or *laissez faire* approach is unlikely to be sufficient for Canadian adaptation to the impacts of climate change.

## Supplementary Files

Supplementary File 1

Supplementary File 2
