# Supplementary material for: Public Health Adaptation to Climate Change in Canadian Jurisdictions
Source: Int J Environ Res Public Health. 2015 Jan 12;12(1):623–51. doi: 10.3390/ijerph120100623 (PMC4306883; doi:10.3390/ijerph120100623)
Supplement: Supplementary File 2 [file ijerph-12-00623-s002.docx]

*Supplementary Materials*

Public Health Adaptation to Climate Change in Canadian Jurisdictions

**Appendix A: Key Terms and Concepts**

The following definitions and descriptions of key terms and concepts guided our analysis and understanding of health adaptation initiatives:

**(1) Vulnerability:**

The following definition of vulnerability from the IPCC guides our understanding of how populations are vulnerable to climate change: “The degree to which a system is susceptible to, or unable to cope with, adverse effects of climate change, including climate variability and extremes. Vulnerability is a function of the character, magnitude, and rate of climate variation to which a system is exposed, its sensitivity, and its adaptive capacity” [1].

**(2) Health Risks:**

A full vulnerability assessment is outside the scope of this study, thus we examine instead the health *risks* posed by climate change addressed by health adaptation initiatives. We focus on any of the following ways health can be affected: food, water, physical harm/injury, illness or death [2]. We categorized health adaptation initiatives as addressing the following health risks posed by climate change, adapted from those identified by the IPCC Working Group II in the 5th Assessment
Report [3]: heat-related, air quality, floods, storms, food security, UV radiation, infectious diseases and general health. The projected or possible health effects of these climate change risks are outlined in the
table below.

We coded discrete health adaptation initiatives addressing infectious diseases as either addressing vector-borne infectious diseases or food- and water-borne infectious diseases, but some data sources did not provide enough information for this distinction. All infectious diseases were thus aggregated to the infectious disease category for analysis. The general health category includes vulnerability assessments, health adaptation initiatives planning a health adaptation strategy, initiatives addressing general emergency preparations, or initiatives intended to protect health as a whole from climate change risks. Examples of initiatives classified as general health include:

- “Raise awareness about health hazards of climate change: The Ministry of Health and
  Long-Term Care established a requirement for increasing public awareness of the health risk factors associated with climate change in the Ontario Public Health Standards and will be undertaking activities to raise awareness in boards of health about this requirement” (Ontario).
- “...providing support through the Northern Strategy Trust Fund to the Northern Climate ExChange to assist three Yukon communities to develop and implement community adaptation plans. ... We will continue to support community-level adaptation planning with technical advice and expertise as well as financial resources” (Yukon).

| **Health Risk** | **Projected/Possible Health Effects** |
| --- | --- |
| Heat-Related Risks | - Heat-related morbidity and mortality - Respiratory and cardiovascular disorders |
| Air Quality | - Eye, nose and throat irritation, and shortness of breath - Acute and chronic damage to the respiratory system - Chronic obstructive pulmonary disease and asthma - Exacerbation of allergies and asthma - Increased risk of cardiovascular diseases  (e.g., heart attacks and ischemic heart disease) - Respiratory and cardiovascular mortality |
| Floods | - Death, injury and illness - Psychological health effects, including mental  health and stress-related illnesses - Health impacts due to food or water shortages - Illnesses related to drinking water contamination - Effects of the displacement of populations  and crowding in emergency shelters - Indirect health impacts from ecological changes,  infrastructure damages and interruptions  in health services |
| Storms | - Death, injury and illness - Psychological health effects, including  mental health and stress-related illnesses - Health impacts due to food or water shortages - Illnesses related to drinking water contamination - Effects of the displacement of populations  and crowding in emergency shelters - Indirect health impacts from ecological  changes, infrastructure damages and  interruptions in health services |
| Food Security | - Impacts on nutrition due to availability of  local and traditional foods from decreases  in ice distribution, stability and duration of  coverage in Northern communities |
| Vector-Borne Infectious Diseases | - Increased incidence of vector-borne infectious diseases native to Canada (e.g., eastern & western equine encephalitis, Rocky Mountain spotted fever) - Introduction of infectious diseases new to Canada - Possible emergence of new diseases, and re-emergence  of those previously eradicated in Canada |
| Food- and  Water-Borne Infectious Diseases | - Sporadic cases and outbreaks of disease from strains  of water-borne pathogenic micro-organisms - Food-borne illnesses - Other diarrheal and intestinal diseases |
| UV Radiation | - Increased risks of skin cancers, eye damage (cataracts), immunosuppression, infectious diseases |

Note: Adapted from Berry *et al*. 2014; Furgal & Séguin, 2006; and Séguin, 2008 [4–6].

**(3) Adaptation Policy:**

The following definition of adaptation policy guides our perspective on adaptation policies and initiatives: “The process leading to the production of outputs in forms of activities and decisions taken by purposeful public and private actors at different administrative levels and in different sectors, which deals intentionally with climate change impacts, and whose outcomes attempt to substantially impact actor groups, sectors, or geographical areas that are vulnerable to climate change” [7]. This definition is particularly helpful in this study focusing on various levels of government health adaptation.

**Appendix B: Inclusion and Exclusion Criteria for Health Adaptation Documents**

For documents to be included in our analysis they had to be a governmental document or website in English or French; have climate change as the overarching focus; address risks posed by climate change to human health; include jurisdictionally relevant initiatives; and be a technical document, adaptation plan, national report, adaptation assessment, vulnerability assessment or government website. The inclusion and exclusion criteria for health adaptation documents are summarized in the table below.

Inclusion and exclusion criteria for health adaptation documents.

| **Inclusion Criteria** | **Exclusion Criteria** |
| --- | --- |
| English or French language | Non-English or French language |
| Climate change as the overarching reasoning | Climate change mitigation |
| Human adaptation to climate change | Non-human adaptation to climate change  (e.g., biodiversity) |
| Jurisdictionally appropriate  documents andinitiatives | Jurisdictionally inappropriate documents andinitiatives (e.g., municipal documents when searching for provincial documents) |
| Government documents, government websites,  or documents by government-established  research organizations/networks or  consultants hired by the government | Documents by non-governmental  organizations, unaffiliated institutions,  private companies or professional associations |
| Technical documents, adaptation  plans,national reports, adaptation  assessments, vulnerability  assessments, government webpages | Editorials, meetings, conferences,  presentations, abstracts, financial  evaluations of climate change adaptation |
| Addresses risks posed to human  health byclimate change (see  Health Risks in Appendix A) | Addresses climate change risks unrelated tohealth (e.g., forest management) |

**Appendix C: Documents or Webpages Included**

Canadian federal, provincial, territorial, regional and municipal adaptation documents or
webpages included

| **Jurisdiction  Name** | **Jurisdiction  Type** | **Author** | **Year** | **Title (URL If Applicable)** |
| --- | --- | --- | --- | --- |
| Canada | Federal | Health Canada | 2007 | About Health Canada(http://www.hc-sc.gc.ca/ ahc-asc/branch-dirgen/hecs-dgsesc/sep-psm/ waccb-beacc-eng.php) |
| Canada | Federal | Health Canada | 2008 | Human Health in a Changing Climate: A Canadian Assessment of Vulnerabilities and Adaptive Capacity |
| Canada | Federal | Natural Resources Canada | 2008 | From Impacts to Adaptation:  Canada in a Changing Climate |
| Canada | Federal | Aboriginal Affairs and Northern Development Canada | 2009 | Implementation Evaluation of INAC Climate Change Adaptation Program: Assist Northerners in Assessing  Key Vulnerabilities and Opportunities (http://www.aadnc-aandc.gc.ca/eng/1307024581995/1307024620213) |
| Canada | Federal | Office of the Auditor General of Canada | 2010 | Report of the Commissioner of the Environment and Sustainable Development to the House of Commons |
| Canada | Federal | Health Canada | 2010 | Whatʼs Being Done about Climate Change and Health in Canada—Adaptive Actions (http://www.hc-sc.gc.ca/ ewh-semt/climat/adapt/actions-eng.php) |
| Canada | Federal | Treasury Board of Canada Secretariat | 2010 | Environment Canada Adaptation Theme  (http://www.tbs-sct.gc.ca/rpp/2010-2011/inst/doe/ st-ts04-eng.asp#Theme:_Adaptation) |
| Canada | Federal | Environment Canada | 2011 | Evaluation of the Improved Climate Change  Scenarios Program (http://www.ec.gc.ca/ ae-ve/default.asp?lang=En&n=4CCFD0E2-1) |
| Canada | Federal | Health Canada | 2011 | Communicating the Health Risks of Extreme  Heat Events: Toolkit for Public Health and  Emergency Management Officials |
| Canada | Federal | Health Canada | 2011 | Extreme Heat Events Guidelines: Technical  Guide for Health Care Workers |
| Canada | Federal | Health Canada | 2011 | Extreme Heat Events Guidelines: User Guide for  Health Care Workers and Health Administrators |
| Canada | Federal | Aboriginal Affairs and Northern Development Canada | 2012 | Climate Change Adaptation Program (http://www.aadnc-aandc.gc.ca/eng/1329158189051/1329158264671) |
| Canada | Federal | Health Canada | 2012 | Heat Alert and Response Systems to  Protect Health: Best Practices Guidebook |

| **Jurisdiction  Name** | | **Jurisdiction  Type** | | **Author** | | **Year** | | **Title (URL If Applicable)** |  |
| --- | --- | --- | --- | --- | --- | --- | --- | --- | --- |
| Canada | | Federal | | Public Health Agency of Canada, Government of Nunavut and Government of Northwest Territories | | 2012 | | NUNAVUT/NORTHWEST TERRITORIES:  Healthy Foods North—A culturally appropriate and community-based program to promote  healthy eating and lifestyle |  |
| Canada | | Federal | | Public Health Agency of Canada | | 2013 | | Preventative Public Health Systems and  Adaptation to a Changing Climate Program (http://www.phac-aspc.gc.ca/hp-ps/eph-esp/ pph-psp-eng.php) |  |
| British Columbia | | Province | | Government of British Columbia | | 2009 | | BC Air Action Plan  (http://www.bcairsmart.ca/) |  |
| British Columbia | | Province | | BC Ministry of Forests, Lands and Natural Resource Operations | | 2012 | | Coastal Flood Hazard Areas In British Columbia (http://www.env.gov.bc.ca/wsd/public_safety/ flood/pdf_drawings/index.html) |  |
| Manitoba | | Province | | Government of Manitoba | | 2008 | | Adapting to Climate Change:  Preparing for the Future |  |
| New Brunswick | | Province | | Government of New Brunswick | | 2007 | | Climate Change Action Plan 2007–2012 |  |
| New Brunswick | | Province | | Government of New Brunswick | | 2014 | | Climate Change Action Plan 2014–2020 |  |
| Newfoundland & Labrador | | Province | | Department of Environment and Conservation | | 2005 | | Climate Change Action Plan |  |
| Newfoundland & Labrador | | Province | | Government of Newfoundland & Labrador | | 2006 | | Charting our Course:  Climate Change Action Plan 2011 |  |
| Newfoundland & Labrador | | Province | | Government of Newfoundland & Labrador | | 2013 | | Enhancing Reslience to Climate Change (http://www.turnbackthetide.ca/government-action/resilience-to-climate-change.shtml) |  |
| Newfoundland & Labrador | | Province | | Department of Environment and Conservation | | 2014 | | Flood risk mapping studies (http://www.env.gov.nl.ca/env/waterres/ flooding/frm.html) |  |
| Northwest Territories | | Territory | | NWT Environment and Natural Resources | | 2008 | | NWT Climate Change Impacts  and Adaptation Report |  |
| Nova Scotia | | Province | | Government of Nova Scotia | | 2005 | | Adapting to a Changing Climate in  Nova Scotia: Vulnerability Assessment  and Adaptation Options |  |
| Nova Scotia | | Province | | Department of Environment | | 2009 | | Toward a Greener Future: Nova Scotia’s  Climate Change Action Plan |  |
| Nova Scotia | | Province | | NS Health and Wellness | | 2012 | | Lyme Disease: A Report on Lyme Disease  Epidemiology and Surveillance in Nova Scotia |  |
| **Jurisdiction  Name** | **Jurisdiction  Type** | | **Author** | | **Year** | | **Title (URL If Applicable)** | | |
| Nunavut | Territory | | Department of Environment | | 2011 | | Upagiaqtavut Setting the Course: Climate  Change Impacts and Adaptation in Nunavut | | |
| Ontario | Province | | Department of Environment | | 2011 | | Climate Ready: Ontario’s Adaptation  Strategy and Action Plan 2011–2014 | | |
| Prairies | Region | | Prairies Adaptation Research Collaborative | | 2001 | | Adaptability of Prairie Cities: The Role of Climate  Current and Future Impacts and Adaptation Strategies | | |
| Prairies | Region | | Prairies Adaptation Research Collaborative | | 2001 | | A Feasibility Assessment to Study Societal Adaptation and Human Health Impacts Under Various Future climate Scenarios Anticipated in the Canadian Prairies | | |
| Prairies | Region | | Prairies Adaptation Research Collaborative | | 2001 | | Isi Askiwan—The State of the Land: Prince Albert  Grand Council Elders’ Forum on Climate Change | | |
| Prince  Edward Island | Province | | Department of Environment | | 2009 | | Prince Edward Island and Climate change: A Strategy for Reducing the Impacts of Global Warming | | |
| Quebec | Province | | Ministère du Développement durable, de l’Environnement et des Parcs | | 2008 | | Quebec and Climate Change: A Challenge for the  Future 2006-2012 Action Plan (Updated) | | |
| Quebec | Province | | Ouranos | | 2008 | | Les effets des changements climatiques  sur la santé au Québec | | |
| Quebec | Province | | Ministère de la Santé et des Services Sociaux | | 2011 | | Changements climatiques: Vulnérabilité  et adaptation des immeubles | | |
| Quebec | Province | | Quebec National institute for Public Health | | 2011 | | SUPREME System  (http://www.ij-healthgeographics.com/content/10/1/39) | | |
| Quebec | Province | | Ministère de la Santé et des Services Sociaux | | 2012 | | Quebec in Action: Greener by 2020—2013–2020 Government Strategy for Climate Change Adaptation | | |
| Quebec | Province | | Government of Quebec | | 2012 | | Quebec in Action: Greener by 2020—2013–2020  Climate Change Action Plan | | |
| Quebec | Province | | Ministere de la Santé et des Services Sociaux | | 2013 | | Plan d'intervention gouvernemental 2013–2015 pour la protection de la population contre le virus du Nil occidental | | |
| Yukon | Territory | | Environment Yukon | | 2009 | | Yukon Government Climate Change Action Plan | | |
| Edmonton, AB | Municipality | | City of Edmonton | | 2011 | | The Way We Green: The City of Edmonton’s Environmental Strategic Plan | | |
| Ottawa, ON | Municipality | | City of Ottawa | | 2004 | | Air Quality & Climate Change Management Plan | | |
| Montreal, QC | Municipality | | Ville de Montréal | | Retrieved  2014 | | Adaptation aux changements climatiques (http://ville.montreal.qc.ca/portal/page?_pageid=7237,75085661&_dad=portal&_schema=PORTAL) | | |

| **Jurisdiction  Name** | **Jurisdiction  Type** | **Author** | **Year** | **Title (URL If Applicable)** |
| --- | --- | --- | --- | --- |
| Ottawa, ON | Municipality | Planning and Growth Department | 2004 | Air Quality & Climate Change Management Plan |
| Toronto, ON | Municipality | City of Toronto | 2011 | Toronto’s Adaptation Actions |
| Vancouver, BC | Municipality | City of Vancouver | 2012 | Climate Change Adaptation Strategy |

**Appendix D: Canadian Federal Departments**

The following five Canadian federal departments are implementing or have implemented health adaptation initiatives: Health Canada, the Public Health Agency of Canada, Aboriginal Affairs and Northern Development Canada, Environment Canada and Natural Resources Canada. We outline the mission, objectives, mandates or visions of these federal departments below.

| **Department Name** | **Description** |
| --- | --- |
| Health Canada (HC) | *Mission*: “Health Canada is the federal department responsible for helping the people of Canada maintain and improve their health” [8].  *Objectives:*   - “Prevent and reduce risks to individual health and the overall environment; - Promote healthier lifestyles; - Ensure high quality health services that are efficient and accessible; - Integrate renewal of the health care system with longer term plans in the areas of prevention, health promotion and protection; - Reduce health inequalities in Canadian society; and - Provide health information to help Canadians make informed decisions” [8]. |
| Public Health Agency of Canada (PHAC) | *Mission:* “To promote and protect the health of Canadians through leadership, partnership, innovation and action in public health” [9].  *Mandate:*   - “Promote health; - Prevent and control chronic diseases and injuries; - Prevent and control infectious diseases; - Prepare for and respond to public health emergencies; - Serve as a central point for sharing Canada’s expertise with the rest of the world; - Apply international research and development to Canada’s public health programs; and - Strengthen intergovernmental collaboration on public health and facilitate national approaches to public health policy and planning” [9]. |

| **Department Name** | **Description** |
| --- | --- |
| Aboriginal Affairs and Northern Development Canada (AANDC) | *Mandate:*  **“**Aboriginal Affairs and Northern Development Canada (AANDC) supports Aboriginal people (First Nations, Inuit and Métis) and Northerners in their efforts to:   - improve social well-being and economic prosperity; - develop healthier, more sustainable communities; and - participate more fully in Canadaʼs political, social and economic development—to the benefit of all Canadians.   AANDC is one of the federal government departments responsible for meeting the Government of Canadaʼs obligations and commitments to First Nations, Inuit and Métis, and for fulfilling the federal governmentʼs constitutional responsibilities in the North” [10]. |
| Environment Canada (EC) | *Mandate:*   - “Preserve and enhance the quality of the natural environment,  including water, air, soil, flora and fauna; - conserve Canadaʼs renewable resources; - conserve and protect Canada's water resources; - forecast daily weather conditions and warnings, and provide  detailed meteorological information to all of Canada; - enforce rules relating to boundary waters; and - coordinate environmental policies and programs for the  federal government” [11]. |
| Natural Resources Canada (NRCan) | “Natural Resources Canada (NRCan) seeks to enhance the responsible development and use of Canada’s natural resources and the competitiveness of Canada’s natural resources products. NRCan develops policies and programs that enhance the contribution of the natural resources sector to  the economy and improve the quality of life for all Canadians” [12].  *Vision*: “Improving the quality of life of Canadians by creating a  sustainable resource advantage” [12]. |

**Appendix E: Canadian Health Adaptation Data**

Accompanying this supplementary materials is an Excel spreadsheet containing the data on discrete health adaptation initiatives used in this study. For each initiative, the jurisdiction, region, document year, implementing agency or body, adaptation type, health risk addressed, adaptation stage, consideration of vulnerble groups and source is provided. Searches and data collection were conducted between February and June 2014, and provincial and territorial searches were conducted again in September 2014.

**Appendix F: Level of Health Adaptation by Province or Territory**

To discern how provinces and territories are progressing in health adaptation compared to one another, we qualitatively assessed their level of health adaptation, shown in Figure 6. We determined the positioning of each province and territory in Figure 6 by creating an index from the number of health adaptation initiatives implemented combined with the percentage of regional risks addressed. Finally, we added “points” to the index for: having a climate change adaptation plan; having a health section in the adaptation plan; the level of detail of the health section and of the health adaptation initiatives; and having information available on climate change and health on their government website. The results are not definitive, but provide an indication for the level of health adaptation in each province and territory. Figure 6 in the paper shows the results of this qualitative ranking.

**References**

1. McCarthy, J.J. *Climate Change 2001: Impacts, Adaptation, and Vulnerability: Contribution of Working Group II to the Third Assessment Report of the Intergovernmental Panel On Climate Change*; Cambridge University Press: Cambridge, UK, 2001.
2. Lesnikowski, A.; Ford, J.; Berrang-Ford, L.; Paterson, J.; Barrera, M.; Heymann, S. Adapting to health impacts of climate change: A study of UNFCCC Annex I parties. *Environ. Res. Lett.* **2011**, *6*, doi:10.1088/1748-9326/6/4/044009.
3. Smith, K.R.; Woodward, A.; Campbell-Lendrum, D.; Chadee, D.D.; Honda, Y.; Liu, Q.;
   Olwoch, J.M.; Revich, B.; Sauerborn, R. Human health: Impacts, adaptation, and co-benefits.
   In *Climate Change 2014: Impacts, Adaptation, and Vulnerability. Part a: Global and Sectoral Aspects. Contribution of Working Group ii to the Fifth Assessment Report of the Intergovernmental Panel of Climate Change*; Field, C.B., Barros, V.R., Dokken, D.J., Mach, K.J., Mastrandrea, M.D., Bilir, T.E., Chatterjee, M., Ebi, K.L., Estrada, Y.O., Genova, R.C., *et al*. Eds.; Cambridge University Press: Cambridge, UK, 2014.
4. Berry, P.; Clarke, K.; Fleury, M.D.; Parker, S. Human health. In *Canada in a Changing Climate: Sector Perspectives on Impacts and Adaptation*; Warren, F.J., Lemmen, D.S., Eds.; Government of Canada: Ottawa, ON, Canada, 2014; pp. 191–232.
5. Furgal, C.; Seguin, J. Climate change, health, and vulnerability in canadian northern aboriginal communities. *Environ. Health Perspect.* **2006**, *114*, 1964–1970.
6. Séguin, J. *Human Health in a Changing Climate: A Canadian Assessment of Vulnerabilities and Adaptive Capacity*; Health Canada: Ottawa, ON, Canada, 2008.
7. Dupuis, J.; Biesbroek, R. Comparing apples and oranges: The dependent variable problem in comparing and evaluating climate change adaptation policies. *Glob. Environ. Chang.* **2013**, *23*, 1476–1487.
8. Health Canada. About Mission, Values, Activities. Available online: http://www.hc-sc.gc.ca/
   ahc-asc/activit/about-apropos/index-eng.php (accessed on 10 September 2014).
9. PHAC. About the Agency. Available online: http://www.phac-aspc.gc.ca/about_apropos/index-eng.php (accessed on 10 September 2014).
10. AANDC. About AANDC. Available online: https://www.aadnc-aandc.gc.ca/eng/1100100010023/
    1100100010027 (accessed on 10 September 2014).
11. Environment Canada. About Environment Canada. Available online: http://www.ec.gc.ca/
    default.asp?lang=En&n=BD3CE17D-1 (accessed on 10 September 2014).

1. Natural Resources Canada. Available online: http//www.nrcan.gc.ca/department (accessed on 10 September 2014).

© 2015 by the authors; licensee MDPI, Basel, Switzerland. This article is an open access article distributed under the terms and conditions of the Creative Commons Attribution license (http://creativecommons.org/licenses/by/4.0/).
